# Fat Malabsorption and Ursodeoxycholic Acid Treatment in Children With Reduced Organic Solute Transporter-α (*SLC51A*) Expression

**DOI:** 10.1097/PG9.0000000000000229

**Published:** 2022-07-25

**Authors:** Rune Rose Tronstad, Siren Berland, Erling Tjora, Khadija El Jellas, Ingvild Aukrust, Kurt Kristensen, Dag Tveitnes, Anders Molven, Hanns-Ulrich Marschall, Anuradha Rao, Paul A. Dawson

**Affiliations:** From the * Department of Pediatrics and Adolescent Medicine, Haukeland University Hospital, Bergen, Norway; † Department of Medical Genetics, Haukeland University Hospital, Bergen, Norway; ‡ Center for Diabetes Research, Department of Clinical Science, University of Bergen, Bergen, Norway; § Gade Laboratory for Pathology, Department of Clinical Medicine, University of Bergen, Bergen, Norway; ∥ Department of Pediatrics, Aarhus University Hospital, Aarhus, Denmark; ¶ Department of Pediatrics and Adolescent Medicine, Stavanger University Hospital, Stavanger, Norway; # Department of Pathology, Haukeland University Hospital, Bergen, Norway; ** Department of Molecular and Clinical Medicine, Sahlgrenska Academy, University of Gothenburg, Gothenburg, Sweden; †† Department of Pediatrics, Emory University School of Medicine, Atlanta, GA.

**Keywords:** 7α hydroxy 4 cholesten 3 one, bile acids, fecal fat, fibroblast growth factor 19

## Abstract

A bile acid homeostasis disorder was suspected in 2 siblings and their second cousin who presented in infancy with fat malabsorption, severe fat-soluble vitamin deficiency, rickets, and mild liver involvement. Our aims were to identify the genetic cause, describe the disease, and evaluate the response to ursodeoxycholic acid (UDCA) treatment.

**Methods:**

Whole exome sequencing, immunohistochemistry of duodenal biopsies and candidate variant testing in a cell-based model was performed. Fecal fat excretion, serum bile acids, 7α-hydroxy-4-cholesten-3-one (C4), and fibroblast growth factor 19 (FGF19) were quantified in both siblings on and off UDCA treatment.

**Results:**

A novel homozygous variant of *SLC51A*, which encodes the bile acid carrier organic solute transporter (OST)-α, was identified in all affected children. OSTα protein expression was readily detected by immunohistochemistry in duodenum of pediatric control subjects but not in the affected siblings. The siblings had low serum levels of bile acids and C4 and high serum levels of FGF19 consistent with repression of hepatic bile acid synthesis. On treatment with UDCA, fecal fat excretion was reduced and serum levels of C4, FGF19, and liver enzymes normalized.

**Conclusions:**

We report an apparent deficiency of OSTα associated with early onset fat malabsorption and mild liver involvement. The clinical presentation partially overlaps previous reports for 3 patients with OSTα or OSTβ deficiency and extends the clinical spectrum associated with loss of *SLC51A* expression. Our data suggest that repression of hepatic bile acid synthesis contributes to fat malabsorption in OSTα-OSTβ deficiency but can be partly reversed with UDCA treatment.

**What Is Known**

- The heteromeric organic solute transporter OSTα-OSTβ (*SLC51A*-*SLC51B*) is essential for intestinal reabsorption and enterohepatic cycling of bile acids.
- Three cases of inherited defects in the OSTα-OSTβ heterodimer have been reported in children with congenital diarrhea and features of cholestasis.

**What Is New**

- Reduced intestinal expression of OSTα protein is reported for 2 new patients who presented with severe fat-soluble vitamin deficiency.
- Plasma markers in the patients indicated reduced hepatic bile acid synthesis.
- Treatment with ursodeoxycholic acid was associated with normalization of serum markers of bile acid synthesis, a decline in fecal fat excretion, and improvement of clinical indices such as growth.

## INTRODUCTION

Inherited defects in bile acid synthesis and transport can cause fat malabsorption and diarrhea (1–3), as well as cholestasis, jaundice and pruritus (4). In the small intestine, bile acids are taken up across the enterocyte brush border membrane by the apical sodium-dependent bile acid transporter (ASBT; also called ileal bile acid transporter; gene symbol, *SLC10A2*) and exported into the portal circulation by the basolateral membrane heteromeric transporter (organic solute transporter α-β [OSTα-OSTβ]) (gene symbols: *SLC51A*, *SLC51B*) (5). Studies in mouse models suggest that blocking bile acid transport at the apical versus basolateral membrane differentially impacts bile acid homeostasis (6,7), and the clinical features associated with ASBT versus OSTα-OSTβ deficiency are predicted to differ. Only 3 cases of disease-causing mutations in OSTα-OSTβ have been reported (8,9). Additional cases must be studied to better understand the spectrum of clinical sequelae, long-term outcomes, and potential therapies for OSTα-OSTβ-related disease, Online Mendelian Inheritance in Man (OMIM) numbers 619484, 619481. In this report, we describe an OSTα deficiency in pediatric patients with fat malabsorption and severe fat-soluble vitamin deficiency, and the impact of treatment with ursodeoxycholic acid (UDCA) on biomarkers of hepatic bile acid synthesis, intestinal fat absorption, and clinical indices, such as growth on full oral nutrition.

## METHODS

### Patients

Two siblings (patients 1 and 2) and their second cousin (patient 3) had similar clinical presentations and were evaluated. All parents received genetic counseling, and informed parental consent was obtained for DNA sequencing, clinical studies, and publication of the findings. Patient 3 and her family now live in a different country, and we were not able to conduct measurements of 7α-hydroxy-4-cholesten-3-one (C4), fibroblast growth factor 19 (FGF19), bile acids, and fecal fat in this subject.

### Genetic Investigation

DNA was isolated from peripheral blood and used for genetic analysis. High density single nucleotide polymorphism (SNP) array Cytoscan HD (Thermo Fischer Scientific) was performed to look for copy number variations (CNVs) and shared long contiguous stretches of homozygosity (LCSH) >5 Mb.

Whole exome sequencing (WES) was performed for the sibling patients (1 and 2) and their parents. DNA was prepared using the SeqCap EZ MedExome Target Enrichment Kit (Roche NimbleGen, Madison, WI) and underwent paired-end 150 nt sequencing on an Illumina NextSeq 500. The paired-end reads were aligned using the Burrows-Wheeler Alignment tool and variant calling was performed using the Genome Analysis Toolkit (Broad Institute, Cambridge, MA) according to Genome Analysis Toolkit’s Best Practices guidelines. Mean reads per base pair in the exome was 91.0 (patient 1) and 86.7 (patient 2), with 98.3% (patient 1) and 97.8% (patient 2) of base pairs covered by at least 10 reads. The specific variant in *SLC51A* was covered by 65 and 80 reads in patients 1 and 2, respectively. Data annotation and interpretation were performed using the Cartagenia Bench Lab, next generation sequencing module (Cartagenia, Leuven, Belgium). The raw data for *SLC51A* exonic sequence including the promoter region and all exonic-intronic boundaries (>90 nt intronic) were also covered by at least 20 reads and were scrutinized for any linked intronic variants. Variant verification by Sanger sequencing (*SLC51A* NM_152672.5) was performed with DNA from all patients.

### Immunohistochemistry of Duodenal Biopsies

Formalin-fixed paraffin-embedded tissue blocks containing duodenal biopsies were sectioned (3–5 µm), deparaffinized in xylene, and rehydrated with decreasing ethanol concentrations.

The paraffin-embedded sections underwent a heat-induced epitope retrieval procedure and were stained using an anti-OSTα antibody (LS-C501191; SBio, Seattle, WA) and EnVision+ (K4003, Agilent Dako) anti-rabbit horse radish peroxidase-conjugated polymer. The sections were counterstained with Mayer’s hematoxylin.

### Site-Directed Mutagenesis and Bile Acid Transport Assays

The human OSTα p.I282T variant was generated using human OSTα in a cytomegalovirus promoter-drive expression plasmid as a template and a Q5 site-direct mutagenesis kit (New England Biolabs, Ipswich, MA). Bile acid transport activity was measured as described (9).

### Fecal Fat Quantification

In each of the 2 study periods, patients collected each stool passed over a 3-day period. Stools were frozen at –20 °C until analyzed. The feces from each collection period were weighed, diluted with water at a 2:1 (w/w) ratio, and homogenized using an Ultra-Turrax@ T50 basic homogenizer (IKA-Werke, Staufen im Breisgau, Germany). The weight amount of fat in each collection period was quantified (10).

### Serum C4 and FGF19 Measurements

Fasting serum samples were collected and used to measure bile acids and C4 by ultra-high performance liquid chromatography with tandem mass spectrometry and FGF19 by ELISA, as previously described (11). FGF19 was analyzed using an FGF19 Quantikine ELISA kit (R&D Systems, Minneapolis, MN), performed in accordance with the manufacturer’s instruction manual.

## RESULTS

### Clinical Description

**FIGURE 1.**
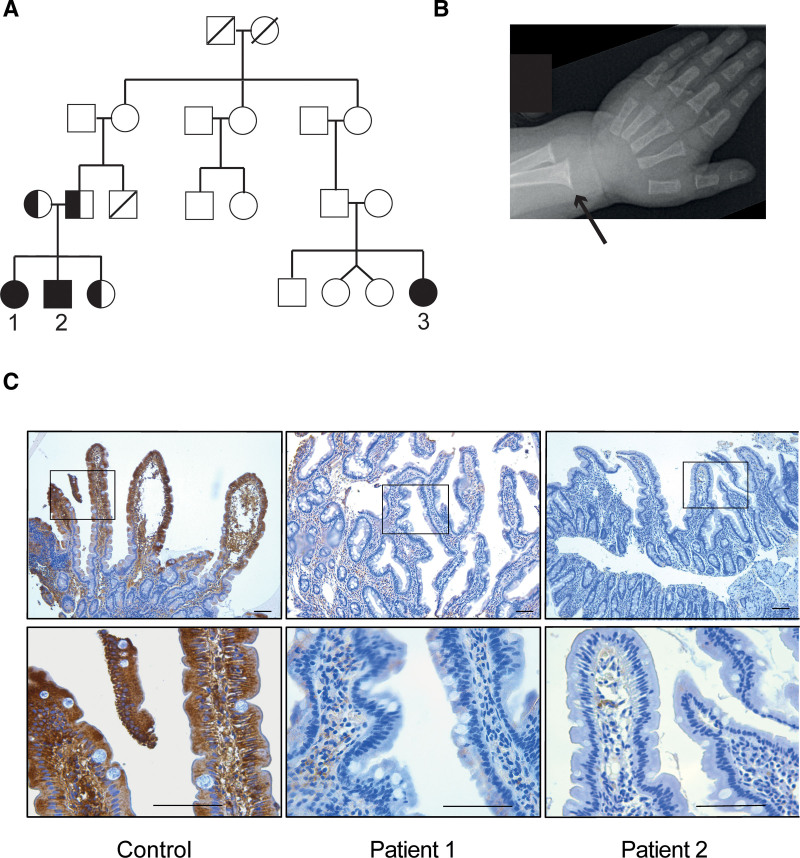
Pedigree, clinical, and histological findings. A) Pedigree of a family with 3 children (1–3) affected by severe fat malabsorption and rickets in infancy. Filled and half-filled symbols indicate homozygosity and heterozygosity, respectively, for the *SLC51A* variant. B) X-ray of left wrist of patient 2 showing distal cupping of ulnar and radial bones (black arrow) typical of rickets. C) Duodenal histology and immunohistochemical detection of OSTα on duodenum tissue biopsies. Histological analysis revealed a variable degree of mucosal injury and a near absence of OSTα staining in the affected siblings. Scale bars are 100 µm in all images. OSTα = organic solute transporter-α.

Patient 1 is the oldest child of parents of Somali origin (Fig. 1A). She was delivered at term with a birthweight of 2080 grams and diagnosed with persistent ductus arteriosus, leading to heart failure, feeding problems, and failure to thrive. At 4 months of age, she underwent surgery, successfully closing the ductus arteriosus. During hospital admission for pneumonia at age 5.5 months, she had up to 3 voluminous stools per day and was still failing to thrive.

She was diagnosed with severe rickets and vitamin A, D, E, and K deficiency (Table 1; vitamin K level was <0.1 ng/mL; range: 0.5–5.0 ng/mL). She was unresponsive to oral supplements of calcium and vitamin D (including alfacalcidol) and was treated with parenteral nutrition and intravenous supplementation of minerals and fat-soluble vitamins.

**TABLE 1.** Clinical and laboratory findings

Clinical chemistry	Reference	Patient 1	Patient 1	Patient 2	Patient 2	Patient 3	Patient 3
UDCA treatment		No	Yes	No	Yes	No	No
Age		5.5 mo	3.5 y	5.5 mo	21 mo	2.5 y	8.5 y
Weight centile		<1st	9th	6th	70th	50th	75th
Bowel movements/d		1–3	2–3	2–3	2–3	4–5	NA
AST (U/L)	15–35	52	45	75	45	NA	NA
ALT (U/L)	10–45	24	18	48	26	33	65
Bilirubin total (mmol/L)	<19	10	6	2	4	NA	NA
GGT (U/L)	10–45	58	9	39	9	NA	NA
Ionized calcium (mmol/L)	1.18–1.37	1.02	1.32	0.76	1.32	0.66	1.25
Vitamin A (µmol/L)	1.2–3.4	0.4	1.5	0.4	1.4	NA	NA
25 OH vitamin D (nmol/L)	50–200	6.9	85	3.4	81	<10	46
Vitamin E (µmol/L)	10.5–43.5	2.7	33	3.0	33	NA	NA
INR	0.9–1.1	NA	NA	1.5	1.0	NA	NA

ALT = alanine aminotransferase; AST = aspartate aminotransferase; GGT = gamma-glutamyl transferase; INR = international normalized ratio; mo = months; NA = not available; UDCA = ursodeoxycholic acid; y = years.

Gastroduodenoscopy with duodenal biopsies revealed villous atrophy and chronic inflammatory changes but no signs of tufting enteropathy or microvillus inclusion disease. Repeat duodenal biopsies after 3 months showed signs of histological improvement with partial villous atrophy and increased intraepithelial lymphocytes. Liver enzymes were moderately elevated (Table 1); however, anti-transglutaminase IgA, fecal elastase, sweat electrolytes, and urinary bile acid levels were normal (data not shown). After a hospital stay of 4.5 months and recovery from rickets, the patient continued with mixed intravenous and partial enteral nutrition (gluten- and cow milk-free, enriched for medium-chain fatty acids). At 2 years of age, UDCA treatment was initiated (20 mg/kg/d), given its safety profile, ability to improve liver function, and potential to promote fat absorption (12–15). Parenteral nutrition was suspended, she transitioned to full oral nutrition, and solid stools were noted. At 3 years of age, she shows normal growth (Table 1) and remains on oral UDCA and fat-soluble vitamins as her only treatment.

Patient 2 is the younger brother of patient 1 and was delivered at term with a normal birthweight of 3030 grams. During admission for a pulmonary infection at 5.5 months of age, he was diagnosed with profound hypocalcemia and vitamin D deficiency. He developed 2 episodes of hypocalcemic convulsions and cardiorespiratory arrest despite ongoing oral supplementation with vitamin D and calcium. Vitamins A and E were low and prothrombin time prolonged, consistent with severe fat-soluble vitamin deficiency (Table 1). Radiography of the left wrist revealed rickets (Fig. 1B). On admission, his weight was at the third centile. He had 2–3 loose, bulky stools per day (Table 1). Liver enzymes (Table 1), sweat electrolytes, fecal elastase, and urine bile acid levels were normal. He required intravenous replacement of fat-soluble vitamins, calcium, and phosphate. Shortly after starting oral UDCA treatment (20 mg/kg/d), he was switched from intravenous to oral mineral and vitamin treatment, and stools were solid with a frequency of only once per day. The boy was discharged after 6 weeks in the hospital at age of 7 months. He is currently showing normal growth and is tolerating full oral feeding with oral UDCA and fat-soluble vitamins as his only treatment. The younger sister of patients 1 and 2 continue to be asymptomatic at 8 months of age.

Patient 3 is the second cousin of patients 1 and 2. She was delivered at term and weighed 3240 grams. Oral vitamin D supplementation was started at 2 weeks of age. At 2.5 months, she was hospitalized due to convulsions and diagnosed with profound hypocalcemia and vitamin D deficiency (Table 1). Her vitamin D deficiency and calcium depletion were initially refractory to oral supplementation but were eventually managed with intramuscular injections of large doses of vitamin D and oral alfacalcidol. She had diarrhea, but normal levels of fecal elastase, and did not respond to oral pancreatic enzyme replacement. An attempt to withdraw alfacalcidol at 6 months of age led to recurrence of hypocalcemia. Her liver enzymes were moderately elevated (See Supplemental Digital Content Figure 1, http://links.lww.com/PG9/A89) but her liver biopsy at age 4 years showed normal tissue as assessed by routine histology. She continued on treatment with alfacalcidol, vitamin D3 and calcium, but was lost to follow-up at age 8. The family was recontacted when she was 13 years old and is now continuing oral alfacalcidol and calcium as her only treatment.

### Genetic Variant Detection

SNP array did not reveal any pathogenic or likely pathogenic CNVs in any of the 3 patients. However, they shared 6 LCSH totaling 38 Mb, indicating consanguinity. A bile acid deficiency was suspected based on the clinical presentation, but none of the LCSHs contained OMIM (https://www.omim.org) genes associated with that phenotype (*HSD3B7*, *AKR1D1*, *CYP7B1*, *AMACR*, *SLC51B*, *SLC10A2*, *ATP8B1*, *ABCB11*, *ABCB4*, *SLCO1B1*, *SLCO1B3*, *SLC10A1*). These genes were also manually checked for small CNVs by SNP array and for heterozygous variants by WES. Trio-based WES for patients 1 and 2 revealed no biallelic or de novo (likely) pathogenic variants in known morbid genes. Further examination of genes potentially relevant for the phenotype and the genome aggregation database (https://gnomad.broadinstitute.org/) revealed a novel homozygous variant (chr3:195959354) in *SLC51A* (NM_152672.5) c.845T>C p.(Ile282Thr) (I282T) (combined annotation dependent depletion tool score of 26.5) as the only match. In addition, we manually scrutinized the raw data on this gene for potential linked variants. *SLC51A* is localized on chromosome 3q29 and included in a shared LCSH of about 15 Mb encompassing a total of 128 protein-coding genes (Chr3[GRCh37]:182424513-197851260). We classified the variant to be of uncertain significance using the ACMG classification system (16) but of potential interest. Sanger sequencing of the *SLC51A* variant confirmed homozygosity for patients 1 to 3 and heterozygosity for the parents and younger sister of patients 1 and 2.

### Immunohistochemistry and Functional Analysis the OSTα I282T Variant

OSTα-OSTβ is expressed along the cephalocaudal axis of the small intestine with the highest levels in the terminal ileum (17). Immunohistochemistry readily detected OSTα protein in age-match control duodenum, with strongest staining in enterocytes near the villus tips. In contrast, duodenal tissue from the affected siblings was almost devoid of OSTα staining (Fig. 1C). To determine whether the OSTα I282T variant affected OSTα expression and/or bile acid transport activity, studies were conducted using virus-transformed monkey kidney cells (COS) transfected with constructs expressing human wild-type OSTβ and either wild-type or variant OSTα. Wild-type OSTα-OSTβ exhibited robust uptake of radiolabeled taurocholate as compared with COS cells transfected with wild-type OSTα plus yellow fluorescent protein expression plasmid (See Supplemental Digital Content Figure 2, http://links.lww.com/PG9/A89). Like wild-type OSTα, OSTα I282T efficiently transported taurocholate when co-expressed with OSTβ in the transfected COS cells.

### Fecal Fat, Markers of Bile Acid Synthesis, and Liver Enzymes Measured On and Off Treatment With UDCA

**FIGURE 2.**
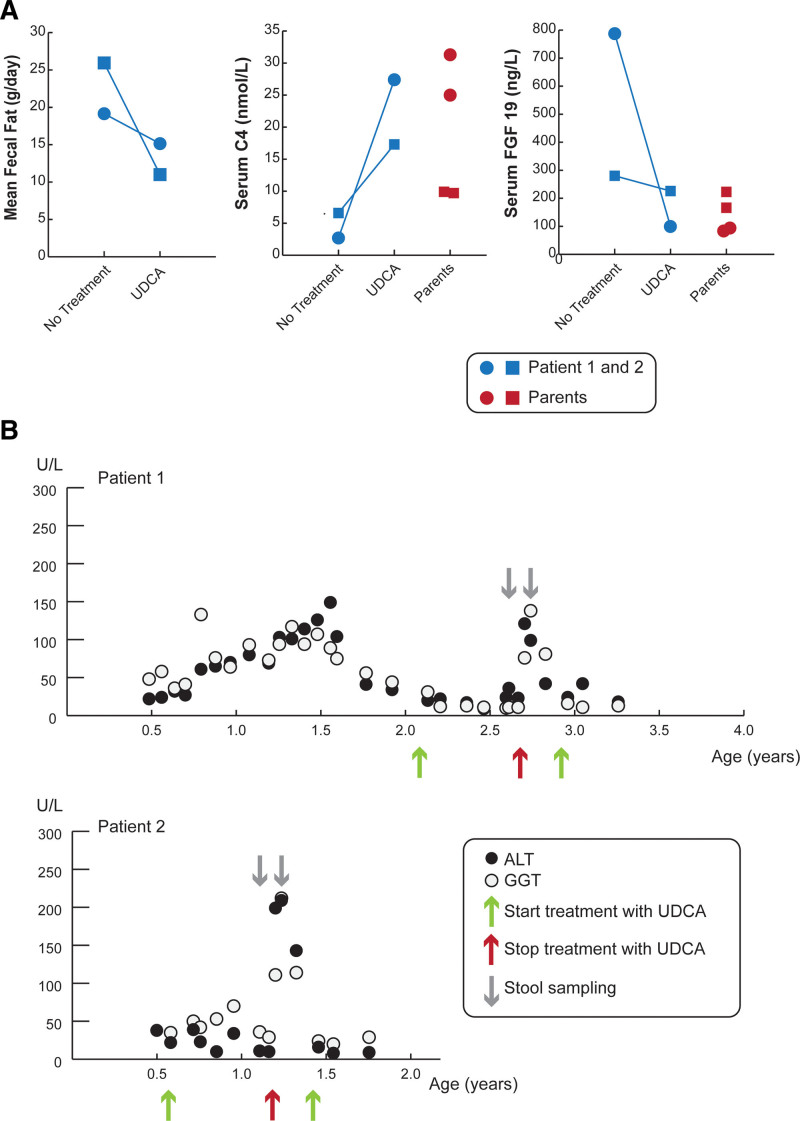
Administration of UDCA reduces fecal fat content, increases serum biomarkers of bile acid synthesis, and normalizes liver enzymes. A) Measurements of daily fecal fat excretion, fasting serum C4, and fasting serum FGF19 off and on treatment with UDCA in patient 1 (blue circle) and patient 2 (blue square). Fasting serum samples were also collected at these times from the mother (red circles) and father (red squares) for measurement of C4 and FGF19. Without UDCA, fasting serum C4 levels were lower and FGF19 levels elevated in the patients versus reference levels in healthy pediatric subjects (reference values, 5th–95th percentile in healthy children: 3.3–134.7 nmol/L for C4 and 33–310 ng/L for FGF19) (18). B) Timeline for the interventions and measurements of ALT and GGT levels in patients 1 and 2 off and on UDCA treatment. At the time of stool sampling (gray arrows), serum was collected to measure bile acids, C4, and FGF19 levels. ALT and GGT levels were normal to moderately elevated over time but showed complete normalization upon treatment with UDCA. ALT = alanine aminotransferase; C4 = 7α-hydroxy-4-cholesten-3-one; FGF19 = fibroblast growth factor 19; GGT = gamma-glutamyl transferase; UDCA = ursodeoxycholic acid.

Off UDCA treatment, fecal fat was elevated in patients 1 and 2. Fasting serum levels of C4 were lower than reference values for healthy children (18), indicative of reduced hepatic bile acid synthesis (Fig. 2A). The reduced C4 levels are potentially secondary to the high serum FGF19 levels, particularly in patient 1. UDCA treatment was associated with reduced fecal fat excretion (Fig. 2A), although steatorrhea (defined as >7 g of fat lost per day) was still present. With UDCA treatment, both C4 and FGF19 normalized into the midrange measured for healthy children. For the parents, the C4 levels for the father were below the 10th percentile, whereas the mother had values near the reference median for healthy adults (19). The FGF19 levels for the mother and father fell within the normal reference range for healthy adults (19), with higher levels in the father. The differences in these values for the affected siblings and the individual parents may reflect variation in the measurements (20). In addition, besides alterations in the enterohepatic bile acid circulation, factors that affect the levels of circulating C4 and FGF19 include the diurnal variation in hepatic bile acid synthesis (21), cholecystectomy (22), and extrahepatic and intrahepatic cholestatic liver disease (23,24). Serum bile acid levels in the affected siblings were 1.6 and 1.2 µM in patients 1 and 2, respectively (reference values, 5th–95th percentiles in healthy children 6–24 months: 6.6–9.4 µM and in healthy children 3–5 years of age: 4.3–6.4 µM) (25) and consisted almost entirely of primary bile acids (See Supplemental Digital Content Table 1, http://links.lww.com/PG9/A89). The secondary bile acid deoxycholate and lithocholate, which are produced by the gut microbiota, accounted for less than 0.01% of the serum bile acids in these patients. This is considerably lower than the fasting serum levels of secondary bile acids normally found in age-matched healthy children (~5%–8%) (25) and is consistent with reduced intestinal absorption of bile acids. The patients exhibited an increase in serum levels of conjugated and unconjugated UDCA on treatment. Levels of the serum liver enzymes alanine aminotransferase and gamma-glutamyl transferase in patients 1 and 2 rose sharply after discontinuation of UDCA and normalized upon reinitiating treatment (Fig. 2B).

## DISCUSSION

We describe 3-related children who presented in infancy with severe fat malabsorption, hypocalcemia, rickets, fat-soluble vitamin deficiency, and mildly elevated liver enzyme levels. Genetic analysis showed that patients were homozygous for a novel missense variant (I282T) in *SLC51A*, the gene encoding OSTα, a basolateral membrane bile acid transporter. The isoleucine residue at position 282 lies in a predicted extracellular loop and is conserved in OSTα orthologs from different species, including xenopus, bovine, mouse, and human (26). We hypothesize that the I282T variant disrupts OSTα-OSTβ in vivo expression, perhaps through protein instability or mislocalization, leading to fat malabsorption in these patients. An undetected linked pathogenic *SLC51A* variant localized deep-intronic or affecting the genomic 3D structure cannot be excluded; however, we have excluded intronic variants in the first 90 nucleotides into the introns, small deletions and promoter variants.

The low C4 and high FGF19 fasting serum levels in these patients with steatorrhea and severe fat-soluble vitamin-deficiency fit predictions from knockout mouse models of OSTα-deficiency (6,27). The inability to induce new hepatic bile acid synthesis coupled with reduced intestinal bile acid reabsorption would lead to gut intraluminal bile acid concentrations insufficient to support efficient lipid digestion and micellization of fatty acids (28,29), cholesterol, and fat-soluble vitamins (2,30–33). This phenotype differs from that observed for bile acid malabsorption caused by partial ileal resection or ASBT-deficiency, where increases in hepatic bile acid synthesis partially compensate for fecal bile acid loss and diarrhea is secondary to an increased bile acid flux into the colon (3,34–37). Although bile acid sequestrants such as cholestyramine may be effective for treating bile acid diarrhea (38), this intervention may worsen clinical outcomes in OSTα-deficient patients by further reducing the amount of intestinal intraluminal bile acids available to participate in fat and fat-soluble vitamin absorption (39,40).

The clinical findings for our patients partially align with those described for 3 previously reported cases of OSTα or OSTβ deficiency (8,9). However, some important differences are also noted, particularly the milder liver and more severe intestinal phenotype in our patients. Possible explanation for the clinical differences includes the nature of the mutation and differential effects on intestinal versus hepatic OSTα expression. The previous reports identified a nonsense (*SLC51A*) and frameshift (*SLC51B*) mutation, which would abolish OSTα-OSTβ function in all tissues. The I282T variant identified in this study did not affect OSTα function when evaluated in a heterologous cell-based assay. As such, it is unclear if or how the I282T variant contributes to the reduced OSTα expression in the duodenal biopsies from these patients. We hypothesize that I282T reduces enterocyte *SLC51A* expression in vivo and the phenotype could not be modeled by expression of the variant-encoding OSTα complementary DNA in COS cells. This hypothesis could be tested using a model such as patient-derived intestinal organoids as well as more detailed analysis of *SLC51A* messenger RNA and protein expression in intestine and liver. Although the bile acid phenotype and response to UDCA closely align with that predicted for an OSTα-OSTβ-deficiency, the presence of an associated biallelic variant in another gene in one of the LCSH cannot be excluded.

Two of the patients were treated with UDCA (41). As a weak detergent, UDCA can promote fat absorption (13) but has a lower lipid solubilizing capacity than chenodeoxycholic acid, cholic acid, or bile acid analogs such as cholylsarcosine (42,43).

However, administration of cholic acid or chenodeoxycholic acid suppresses endogenous hepatic bile acid synthesis (14,44,45), which is already low in these patients. In contrast, UDCA and its glycine conjugate can act as a farnesoid X-receptor antagonist in humans (15,45) to stimulate endogenous hepatic bile acid synthesis (45,46), which is predicted to be beneficial for promoting intestinal lipid absorption. On UDCA treatment, biomarkers of endogenous bile acid synthesis were elevated, fecal fat loss was reduced, liver enzymes normalized, diarrheal symptoms resolved, and the siblings were able to suspend intravenous vitamins and minerals and gradually reduce oral supplements. These findings align with animal studies demonstrating that the fat malabsorption associated with OSTα-deficiency can be reversed by increasing gut intraluminal concentrations of bile acids (47). UDCA was used in the previous reported case of OSTα deficiency (8). That patient’s coagulopathy resolved, and the number of bowel movements per day decreased following treatment with UDCA and cholestyramine, supporting a role for UDCA in this disorder. However, his liver enzyme levels remained elevated, potentially reflecting a more detrimental (loss-of-function) genotype. Further investigation of additional subjects will be needed to understand the contribution of decreased bile acid synthesis to the clinical presentation of OSTα-OSTβ deficiency.

In conclusion, we report 3 children with suspected inheritable dysfunction of the α-subunit of OSTα-OSTβ. The clinical presentation partially overlaps with 3 previously reported cases of OSTα-OSTβ deficiency and highlights potential genotypic and environmental differences in the presentation, since the particularly severe vitamin D deficiency may have been precipitated due to scarce sunlight in northern latitudes where our patients of Somali descent are living (48). Our findings suggest that reduced hepatic bile acid synthesis contributes to the clinical presentation and that UDCA has therapeutic benefit for this rare disorder by directly promoting lipid absorption and stimulating endogenous hepatic bile acid synthesis.

## ACKNOWLEDGMENTS

We thank Ingeborg Brønstad, Department of Clinical Medicine, University of Bergen for assistance with the fat quantification and the Department of Radiology, Haukeland University Hospital, Bergen for diagnostic imaging. We dedicate this article to the memory of Dr Torunn Fiskerstrand, dear mentor and inspirator, who sadly passed away shortly after embarking on this project.

R.R.T. involved in planning the study, collecting and interpreting the data, writing the first draft of the article, and critically reviewing the article. S.B., E.T., and H.-U.M. involved in planning the study, collecting and interpreting the data, and critically reviewing the article. K.E.J., I.A., K.K., A.M., and A.R. involved in collecting and interpreting the data and critically reviewing the article. D.T. involved in collecting the data and critically reviewing the article. P.A.D. involved in interpreting the data, editing the article, and critically reviewing the article.

## Supplementary Material


