# Supplementary material for: Fat Malabsorption and Ursodeoxycholic Acid Treatment in Children With Reduced Organic Solute Transporter-α (SLC51A) Expression
Source: JPGN Rep. 2022 Jul 25;3(3):e229. doi: 10.1097/PG9.0000000000000229 (PMC9491403; doi:10.1097/PG9.0000000000000229)
Supplement: Supplementary file 1 [file pg9-3-e229-s001.pdf]

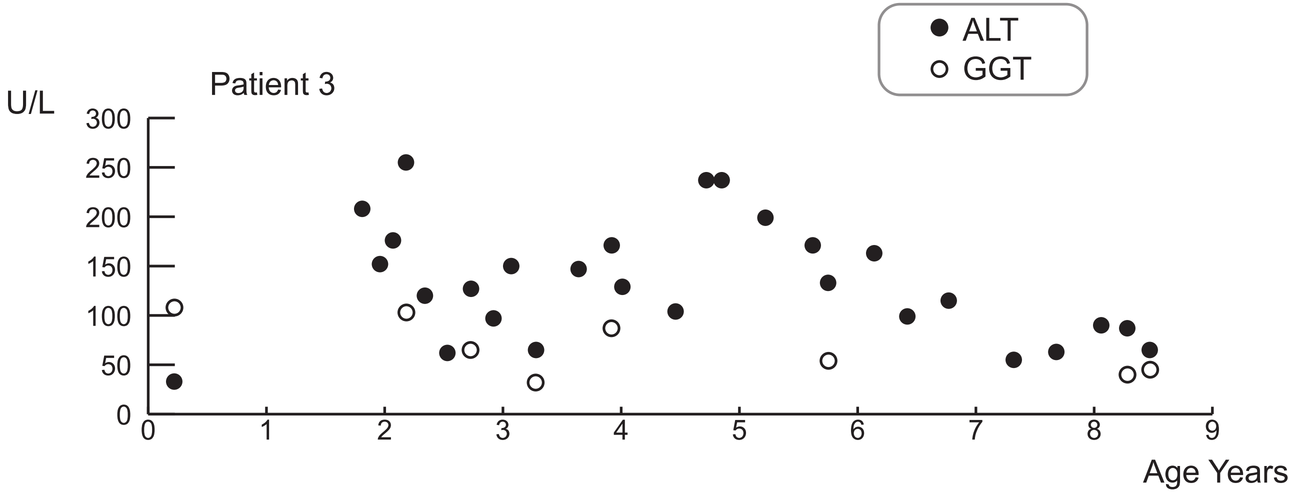

ALT
GGT
Patient 3
U/L
300
250
200
150
100
50
0
0
1
2
3
4
5
6
7
8
9
Age Years

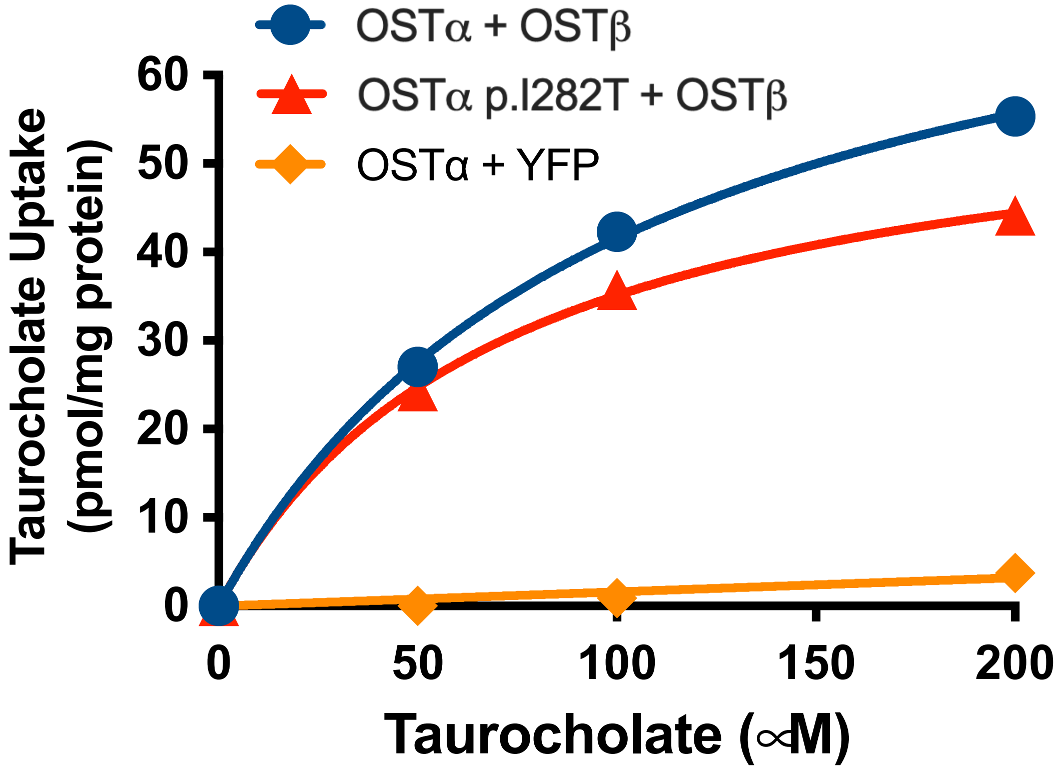

OSTα + OSTβ
OSTα p.I282T + OSTβ
OSTα + YFP
60
50
40
30
20
10
0
Taurocholate Uptake (pmol/mg protein)
0
50
100
150
200
Taurocholate (∝M)

Supplemental Table 1. Serum Bile Acids

Bile Acid (nmol/L)	Patient 1	Patient 1 UDCA	Patient 2	Patient 2 UDCA
TCA	232.8	8.7	229.0	16.1
GCA	155.1	56.3	582.9	90.3
TCDCA	200.1	10.5	55.0	10.7
GCDCA	366.0	171.4	222.2	157.1
T/G DCA	0.0	0.0	0.0	0.0
T/G LCA	0.0	0.4	0.4	1.3
CA	49.5	64.5	13.7	128.9
CDCA	387.5	358.7	53.3	264.1
DCA	0.7	0.5	0.8	1.0
HCA	110.4	4.2	33.2	14.8
TUDCA	2.7	35.1	0.3	19.3
GUDCA	14.5	1751.0	7.1	1365.6
UDCA	13.8	1761.3	2.6	1017.8
iso-UDCA	57.1	392.3	22.3	488.0
Other	19.9	36.5	8.3	17.1
Total	1610	4647	1198	3577

Serum bile acid species in patients off and on UDCA treatment. The concentration of taurine (T) and glycine (G) conjugated and unconjugated bile acids are shown as nmoles per liter. Minor bile acid species, which include HDCA, MDCA, αMCA, βMCA, ωMCA are summed and listed as Other. Cholic acid (CA), chenodeoxycholic acid (CDCA), deoxycholic acid (DCA), hyodeoxycholic acid (HCA), ursodeoxycholic acid (UDCA), hyodeoxycholic acid (HDCA), murideoxycholic acid (MDCA), muricholic acid (MCA)
